# BET and CDK Inhibition Reveal Differences in the Proliferation Control of Sympathetic Ganglion Neuroblasts and Adrenal Chromaffin Cells

**DOI:** 10.3390/cancers14112755

**Published:** 2022-06-01

**Authors:** Jessica Sriha, Caroline Louis-Brennetot, Cécile Pierre-Eugène, Sylvain Baulande, Virginie Raynal, Amira Kramdi, Igor Adameyko, Uwe Ernsberger, Thomas Deller, Olivier Delattre, Isabelle Janoueix-Lerosey, Hermann Rohrer

**Affiliations:** 1 Institute of Clinical Neuroanatomy, Dr. Senckenberg Anatomy, Neuroscience Center, Goethe University, 60590 Frankfurt am Main, Germany; jessica.sriha@tron-mainz.de (J.S.); ernsberger@outlook.de (U.E.); t.deller@em.uni-frankfurt.de (T.D.); 2 Inserm U830, Diversity and Plasticity of Childhood Tumors Laboratory, PSL Research University, SIREDO Oncology Center, Institut Curie Research Center, 75005 Paris, France; caroline.louis@curie.fr (C.L.-B.); cecile.pierre-eugene@curie.fr (C.P.-E.); amira.kramdi@curie.fr (A.K.); olivier.delattre@curie.fr (O.D.); isabelle.janoueix@curie.fr (I.J.-L.); 3 NGS Platform, Institut Curie, CEDEX 05, 75248 Paris, France; sylvain.baulande@curie.fr (S.B.); virginie.raynal@curie.fr (V.R.); 4 Department of Neuroimmunology, Center for Brain Research, Medical University of Vienna, 1090 Vienna, Austria; igor.adameyko@ki.se; 5 Department of Physiology and Pharmacology, Karolinska Institutet, SE-17177 Stockholm, Sweden

**Keywords:** chromaffin cell, sympathetic, neuroblast, bromodomain and extraterminal (BET) protein, transcriptional cyclin-dependent kinase

## Abstract

**Simple Summary:**

Neuroblastoma is a childhood tumor of the sympathetic nervous system. Abdominal tumors that arise in the adrenal differ genetically and clinically from tumors that develop from sympathetic ganglia. Adrenal chromaffin cells and ganglionic neuroblasts are derived from different lineages, raising the possibility that their diverse tumor characteristics are due to different tumor founder cells. To identify traits that are specific to the candidate founder cells of adrenal and ganglionic tumors, cultures of mouse chromaffin cells and sympathetic neuroblasts were treated with a panel of proliferation inhibitors that affect various signaling pathways. Transcription-related inhibitors (BET, CDK7/12/13) showed differential effects, indicating that the BET protein and CDK signaling pathways differ in their relevance for proliferating chromaffin cells and sympathetic neuroblasts. Thus, the oncogenic aberrations affecting these pathways should differ in their efficiency and result in selective propagation, which may lead to adrenal and ganglionic tumors having different characteristics.

**Abstract:**

Neuroblastoma arising from the adrenal differ from ganglionic neuroblastoma both genetically and clinically, with adrenal tumors being associated with a more severe prognosis. The different tumor properties may be linked to specific tumor founder cells in adrenal and sympathetic ganglia. To address this question, we first set up cultures of mouse sympathetic neuroblasts and adrenal chromaffin cells. These cultures were then treated with various proliferation inhibitors to identify lineage-specific responses. We show that neuroblast and chromaffin cell proliferation was affected by WNT, ALK, IGF1, and PRC2/EZH2 signaling inhibitors to a similar extent. However, differential effects were observed in response to bromodomain and extraterminal (BET) protein inhibitors (JQ1, GSK1324726A) and to the CDK-7 inhibitor THZ1, with BET inhibitors preferentially affecting chromaffin cells, and THZ1 preferentially affecting neuroblasts. The differential dependence of chromaffin cells and neuroblasts on BET and CDK signaling may indicate different mechanisms during tumor initiation in sympathetic ganglia and adrenal.

## 1. Introduction

The sympathetic nervous system, which is composed of neurons in the sympathetic ganglia and chromaffin cells of the adrenal medulla, is essential for the control of body homeostasis [1]. Ganglionic neurons and adrenal chromaffin cells were initially thought to arise from a common sympathoadrenal progenitor whose fate is specified by signals from the environment [2,3]. In vivo lineage tracing and single cell RNA (scRNA) sequencing data from mouse sympathoadrenal cells subsequently suggested that sympathetic neurons and chromaffin cells are derived from different progenitors, neural crest cells, and Schwann cell precursors (SCPs), respectively [4]. This scheme has recently been refined by the demonstration that the SCP-derived lineage also includes the neuroblasts located in mouse and human adrenal medulla [5,6,7,8,9].

Understanding sympathoadrenal development is essential for the analysis and comprehension of neuroblastoma development, a childhood tumor that arises from founder cells in the adrenal medulla and sympathetic ganglia [10,11]. Neuroblastoma located in the sympathetic ganglia and adrenal tumors differ clinically and genetically in that adrenal tumors are more aggressive and have a worse prognosis than ganglionic tumors. Adrenal tumors more often display segmental chromosomal aberrations (SCAs), including *MYCN* amplifications, compared to ganglionic tumors, which exhibit rather numerical chromosomal aberrations [12,13,14]. The distinct properties of adrenal and ganglionic tumors may be explained by either the selective signals acting on common progenitors or by the distinct tumor founder cells that differ in their vulnerability to oncogenic drivers. Recently, several studies have addressed the cellular origin of neuroblastoma by comparing normal medullary cells from embryonic mouse and human adrenal with malignant neuroblastoma cells using scRNA sequencing to define the cell identity [6,7,8,9]. Neuroblastomas were found to be mainly composed of tumor cells that resemble normal adrenal neuroblasts, implicating neuroblasts rather than chromaffin cells as neuroblastoma founder cells.

However, the proposed projections from normal developing cells to malignant cells rely on the fundamental premise that the original signature of the founder cell is maintained during tumor initiation and progression [15]. In view of the developmental plasticity of the chromaffin cells and neuroblasts observed both in vivo and in vitro [7,16,17,18], adrenal tumor development may involve chromaffin cells that shift towards a neuroblast identity during tumor initiation and progression. In this scenario, the high frequency of the SCAs and *MYCN* amplifications in adrenal tumors would be caused by different sensitivities of chromaffin cells and neuroblasts to oncogenic signals. To address these issues, we developed a cell biological approach to characterize candidate founder cell populations in the adrenal and sympathetic ganglia of wild-type mice.

Here, we set up cultures of proliferating chromaffin cells from postnatal mouse adrenal and sympathetic neuroblast cultures from paravertebral ganglia to identify the signaling pathways involved in their proliferation. NESTIN-expressing adrenal cells served as non-neuronal and non-adrenergic controls [19,20]. We focused on the ALK, IGF1, WNT, EZH2/PRC2, BET, CDK7, and CDK12/13 signaling pathways since they have been shown to be important during the normal development of sympathoadrenal cells and/or involved in neuroblastoma tumorigenesis.

The activating mutations in the anaplastic lymphoma kinase gene (*ALK*) play important roles in familial and sporadic neuroblastoma [21,22,23,24]. Sympathetic neuroblasts respond to activated ALK with increased proliferation followed by neuron differentiation, but tumor induction requires a combination of activated ALK and MYCN overexpression or massive ALK over-expression [25,26,27,28,29,30]. ALK mutations are enriched in thoracic compared to adrenal neuroblastoma [14], which may be caused by stronger dependency of neuroblasts on ALK signaling, in line with higher ALK expression in mouse and human neuroblasts compared to chromaffin cells [4,6,9]. Sympathetic neuroblast proliferation was shown to be controlled by ALK signaling in vitro, but its function in chromaffin cell cultures has not been studied yet [29,30].

The IGF signaling pathway is important for embryonic sympathetic neuroblast proliferation and for neuroblastoma development [31,32]. Indeed, interfering with IGF signaling inhibits neuroblast proliferation and neuroblastoma growth both in vitro and in vivo [31,33]. Sympathetic neuroblast proliferation also depends on the canonical WNT pathway involving FZD/β-CATENIN [34]. However, the role of WNT/β-CATENIN signaling in neuroblastoma is unclear as over-activation and inhibition both interfere with the survival and proliferation of neuroblastoma cell lines [35]. In addition, different cell lines respond either by proliferation or differentiation to WNT activation [36]. Neuroblastoma cell lines also demonstrate heterogenous responses to IGFR-I targeting agents, raising the issue whether neuroblast and chromaffin cell proliferation may depend differently on IGF and WNT signaling [37].

Epigenetic mechanisms are likely involved in the development of pediatric tumors, including neuroblastoma [38]. The polycomb repressor complex 2 (PRC2) with histone methyltransferase EZH2 as a key component represses gene expression during development and has been implicated in the epigenetic control of many adult cancers [39]. EZH2 is highly expressed in MYCN-amplified neuroblastoma, maintaining the tumor cells in an undifferentiated state, and EZH2 inhibition reduces neuroblastoma growth in vitro and in vivo [40]. Interestingly, neuroblastoma initiation in TH-MYCN mouse models involves sustained EZH2 expression and the suppression of PRC2 targets in MYCN-expressing sympathetic neuroblasts [41]. The importance of PRC2 in MYCN-induced tumor formation is shown by the suppression of tumor development in response to EZH2 inhibition [41]. Whether interfering with PRC2/EZH2 affects the growth of normal ganglionic neuroblasts or chromaffin cells, as implicated by high EZH2 expression, has not been studied.

Super-enhancers control lineage-specific gene expression programs and are frequently deregulated in cancers, including neuroblastoma [42,43]. Aberrant enhancer programs can be targeted epigenetically by BET protein inhibition or by interfering with the function of transcription-related cyclin-dependent kinases (CDKs) [44,45,46]. BET and CDK-7 inhibition display selective effects on MYCN-amplified tumor cells, which may be explained by preferentially targeting super-enhancer driven transcription [44,46,47]. The growth inhibitory effect of the BET inhibitor JQ1 is, however, also observed in cultured normal sympathetic neuroblasts [30]. The effect of BET inhibition in cancer cell lines could also be linked to the functions of BET proteins in the prevention of transcription–replication conflicts and DNA damage [48,49,50]. As sympathetic neuroblasts and chromaffin cells belong to different lineages and differ in terms of MYCN expression, it is of interest to investigate whether they show different vulnerability to BET and CDK inhibition.

A prominent feature of developing chromaffin cells is their ability to transdifferentiate into a neuroblast/neuron identity in response to various environmental signals [16,18,51,52]. This is why we analyzed the extent to which chromaffin cells maintain their identity under the present culture conditions.

## 2. Materials and Methods

### 2.1. Cultures of Adrenal Chromaffin Cells and Adrenal NESTIN-Expressing Cells

#### 2.1.1. Cell Preparation

Animals were killed in accordance with the German law on animal welfare (Tier-schutzgesetz). Adrenal cells were dissected from postnatal OF-1 mice (P1–P5) (Charles River, Sulzfeld, Germany), collected in PBS/glucose (1 mg/mL) on ice, and cut into 2–4 pieces using feather scalpels. The tissue was transferred to 1 mL papain/DNAse solution (Worthington Papain LS003126 0.6 Units/mL; DNAse 0.04 mg/mL in PBS/Glucose) and digested for 12 min at 37 °C. After collection by centrifugation, the adrenal cells were re-suspended in 1 mL Dispase/collagenase/DNAse (Worthington CLS-1 17 U/mg; Dispase II Roche 0.06 U/mg; DNAse 0.04 mg/mL) in PBS/glucose and digested for an additional 10 min. After centrifugation (300 g/3 min) and re-suspension in 1 mL PBS/glucose plus 0.2% BSA, the tissue was dissociated to single cells by trituration using a siliconized, fire-polished Pasteur pipette. The suspension was filtered through a 40 μm cell strainer, collected by centrifugation, and re-suspended in complete serum-free medium (Ham’s DMEM/F12 (Sigma-Aldrich, Taufkirchen, Germany) supplemented with N2 (1%), B27 (2%), glutamine, penicillin/streptomycin, EGF (20 ng/mL), FGF (20 ng/mL) (all from Gibco/Thermofisher, Dreieich, Germany), and heparin (Sigma 43149; 10 U/mL). Differential plating was used to reduce the number of adrenocortical and endothelial cells by maintaining the cells overnight in 10 cm Corning ultra-low attachment dishes at a density of about 1 × 10^6^ cells/mL [20]. To eliminate cell debris, density step gradient centrifugation was carried out, where the cell suspension was overlayed onto 2 mL DMEM/F12 with 3% BSA in a 15 mL Falcon tube. After centrifugation (300 g, 6 min), the pellet was suspended in 1 mL PBS/0.02% BSA.

#### 2.1.2. Immunopanning

First, 10 cm Petri dishes were coated with 10 μg/mL goat anti-rat IgG + IgM (Jackson Immuno Research 112-005-044) in 50 mM Tris/HCl pH 9.5 (100 μg/dish) for 24 h at 4 °C. After being washed two times with PBS, the dish coated with anti-rat IgG + IgM was incubated with 0.2 ug/mL rat anti-L1CAM antibody (clone 324, MAB5272, Millipore, Darmstadt, Germany) for 24 h at room temperature. After being washed twice with PBS, 9 mL PBS/0.02% BSA was added. After the density gradient, the adrenal cells (1 mL in PBS/0.02% BSA) were added to the anti-L1CAM-coated dish and incubated for 90 min at room temperature. The dish was then flushed repeatedly to remove all the cells that did not firmly attach to the anti-L1CAM dish (L1CAM-negative cell fraction). The attached cells (L1CAM-positive) were harvested by digestion with trypsin (Worthington TRL3 Trypsin, 180 U/mL in Earles Balanced Salt Solution) for 8–10 min at 37 °C and 5% CO_2_. The trypsin treatment was stopped by the addition of trypsin inhibitor (Worthington Soybean Trypsin inhibitor). Both L1CAM-negative cells and the L1CAM-positive cells were collected by centrifugation (6 min/300 g) and were re-suspended in complete serum-free medium.

#### 2.1.3. Sphere Cultures

L1CAM-positive and L1CAM-negative cells were plated in non-adherent 35 mm 6-well-plates (Corning, VWR, Darmstadt, Germany) at a density of 100.000 cells/mL and maintained in complete serum-free medium (37 °C, 5% CO_2_). For histological analysis, the spheres were plated on culture dishes coated with poly-DL-ornithine (Sigma) and laminin (Invitrogen, Darmstadt, Germany) overnight followed by fixation with 4% paraformaldehyde and immunostaining. For RNA sequencing, the spheres were collected by centrifugation. Cells were lysed with the addition of 350 μL RA1 lysis buffer (NucleoSpin RNA kit, Macherey Nagel, Düren, Germany) and 35 μL β-mercaptoethanol and were then processed for RNA isolation.

Adherent cultures: L1CAM-positive and L1CAM-negative cells were plated overnight on 35 mm 4-well-dishes (Greiner, Frickenhausen, Germany) coated with poly-DL-ornithine and laminin (5–10,000 cells in 80 μL per well) [30]. The following day, medium was adjusted to a final volume of 1.5 mL/dish, and inhibitors were added.

### 2.2. Sympathetic Neuroblast Cultures

#### Cell Preparation

Animals were killed in agreement with the German law on animal welfare (Tier-schutzgesetz). Sympathetic ganglia (superior cervical ganglia (SCG), stellate ganglia (STG), and thoracic ganglia) were dissected from E14.5 mouse embryos. Dissociation followed the protocol described for postnatal adrenals with reduced protease treatment (7 min and 6 min for papain and collagenase/dispase, respectively). After density step gradient centrifugation, the cells were suspended in serum-free medium and plated on porn/laminin-coated 4-well dishes (80 μL/well) at a density of 5–8000 cells/well. The following day, the medium was adjusted to a final volume of 1.5 mL/dish, and inhibitors were added. The culture conditions for the neuroblasts were slightly modified to increase substrate attachment compared to the adrenal-derived cells. In brief, Ham’s DMEM/F12 (Sigma) was supplemented with N2 (1%), B27 (2%), glutamine, penicillin/streptomycin, IGF (20 ng/mL), FGF (20 ng/mL), β-mercaptoethanol (55 μM), and 2% chick embryo extract (modified from [53]). To prevent the neuroblast clusters from detaching, the culture period was restricted to 3 days.

### 2.3. Immunostaining and Proliferation Analysis

#### 2.3.1. Immunostaining

Immunostaining was carried out as described in detail previously [54]. The antibodies used for adrenal cell and neuroblast characterization were mouse anti-TH (generated and characterized previously [55]), rabbit anti-TH (Ab 152; Merck, Darmstadt, Germany), goat anti-PHOX2B (R&D, Wiesbaden, Germany; AF4940SP)), mouse anti-NESTIN (Sigma MAB353), chicken anti-Nestin (AvesLabs, Davis, CA, USA), rat anti-L1CAM clone 324 (Millipore MAB5272), mouse anti-VMAT1 (Santa Cruz, Heidelberg, Germany; sc-166391), mouse anti-PNMT (Santa Cruz sc-393995), mouse anti-DLK1 (Santa Cruz sc-376755), mouse anti-SGII (Santa Cruz sc-53441), rabbit anti-CHGA (Novus, Cambridge, UK; NB120-15160SS), rabbit anti-PERIPHERIN (Abcam, Cambridge, UK; ab246502), mouse anti-TUBB3 (HISS Diagnostics, Freiburg, Germany; MMS 435P), mouse anti-S100B (Sigma S2532), mouse anti-STAR (Santa Cruz sc-166821), and mouse anti-SOX10 (gift from Michael Wegner, Erlangen, Germany). Mouse anti-ISLET-1 (39.4D5) was obtained from the Developmental Studies Hybridoma Bank, Iowa, IA, USA. The secondary antibodies used were goat anti-rabbit IgG Alexa 488 (Thermofisher A11035), donkey anti-goat IgG Alexa 568 (Thermofisher A11057), donkey anti-mouse IgG Alexa 488 (Thermofisher A21202), goat anti-mouse IgG Alexa 488 (Thermofisher A11001), goat anti-rabbit IgG Alexa 594 (Thermofisher A10037), donkey anti-rabbit IgG Alexa 488 (Thermofisher A21206), and donkey anti-rabbit IgG Alexa 568 (Thermofisher A10042). For quantification, 300–400 cells were analyzed for the expression of specific antigens. The percentage of antigen-positive cells is the mean ± standard error of the mean (s.e.m). of at least three independent culture experiments.

#### 2.3.2. Proliferation Analysis and Pharmacological Treatments

Inhibitors were dissolved in Dimethylsufoxide (DMSO) at 2–5 mM, diluted to appropriate final concentrations, and added on day one of the culture period. The inhibitors that were used were the BET inhibitors JQ1 (Tocris Biotechne, Wiesbaden, Germany; 4499) and GSK1324726A (iBET 726) (Selleckchem Biozol, Eching, Germany), the CDK-7 inhibitors THZ1 (Medchem Express Biotrend, Köln, Germany) and YKL-5-125 (Selleckchem), the CDK12/13 inhibitor THZ 531 (Selleckchem), the IGF1-R inhibitor picropodophyllin (PPP) (Tocris 2956), the EZH2 inhibitor EPZ 6438 (Axon Medchem, Groningen, NL; 2227), the WNT inhibitor ICG001 (Axon Medchem 1766), and the ALK inhibitor Alectinib (Selleckchem S276).

Cell proliferation was analyzed for chromaffin cells and NESTIN-expressing cells after 6 days in culture and for neuroblasts after 3 days in culture by 4 h 5-ethynyl-2-deoxyuridine (EdU) labeling using the Click-iT EdU Alexa Fluor 594 imaging kit (Invitrogen Thermofisher, Dreieich, Germany) combined with staining for PHOX2B and NESTIN. The effects of the BET and CDK inhibitors on the chromaffin cells were also analyzed in the neuroblast-medium after a 3d culture period.

### 2.4. RNA Extraction and Sequencing

Total RNA was extracted from frozen cells using the NucleoSpin RNA kit (Macherey-Nagel). Samples were subjected to quality control on a bioanalyzer instrument and only RNA with RIN (RNA Integrity Number) > 6 were used for sequencing. RNA sequencing libraries were prepared from 500 ng of total RNA using the Illumina TruSeq Stranded mRNA Library preparation kit (Illumina, Evry, France), which allows strand-specific sequencing to be performed. Sequencing (Paired-end 50) was performed with the Illumina HiSeq2500 instrument.

Reads were aligned to the mouse reference genome mm10 using STAR 2.6.1a_08-27 with the default parameters and—outFilterMismatchNoverLmax 0.04 [56]. Gene expression values (FPKM = fragments per kilobase per million reads) were computed by Cufflinks v2.2.146 [57], and further normalization between samples was carried out using quantile normalization (R/Bioconductor package LIMMA) [58].

### 2.5. Statistical Analysis

Data are displayed as mean ± s.e.m. from at least three biological replicates from independent experiments. Differences were analyzed using two-tailed unpaired *t*-tests using the GraphPad Prism software. *p*-values less than 0.05, 0.01, and 0.001 were assigned as being statistically significant (*, **, and ***).

## 3. Results

### 3.1. Characterization of Sympathetic Neuroblasts and Adrenal Chromaffin Cells

In sympathetic ganglia, neuroblast proliferation is restricted to embryonic stages and terminates at E17.5 in the mouse [59]. In contrast, rodent adrenal chromaffin cells continue to proliferate at postnatal stages [17,60,61]. Thus, embryonic neuroblasts and postnatal chromaffin cells were used to investigate the signaling mechanisms involved in their proliferation.

**Figure 1:**
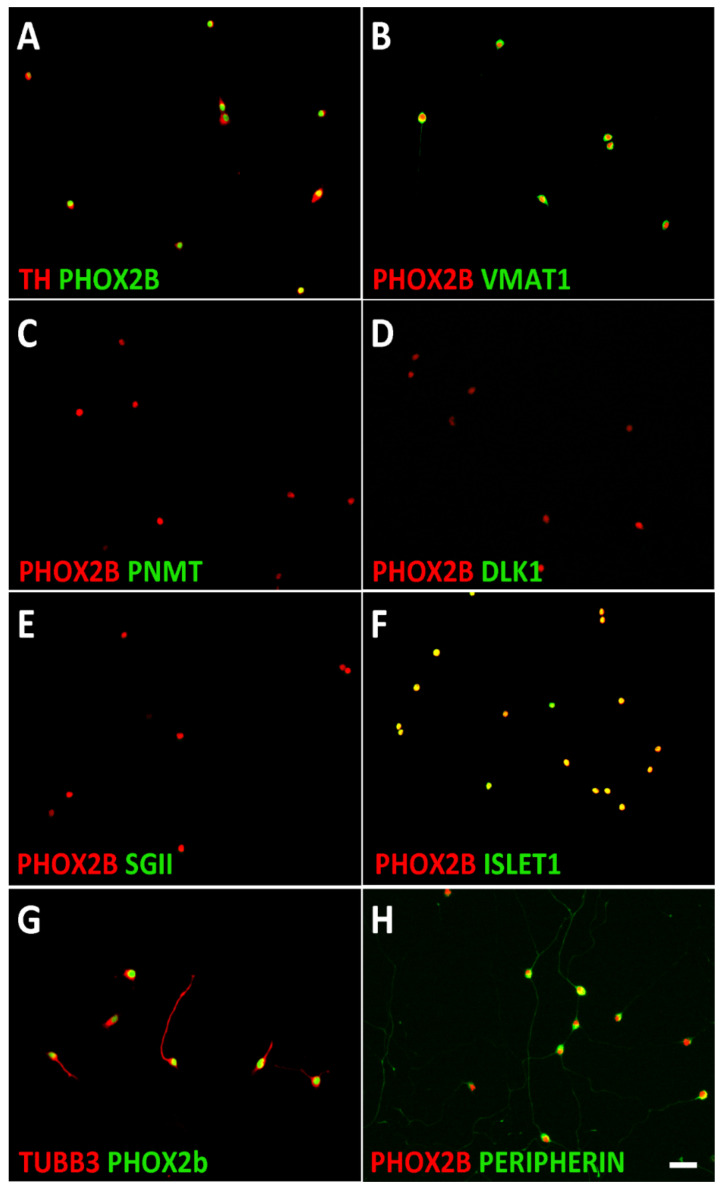
Characterization of mouse sympathetic neuroblasts. Dissociated E14.5 sympathetic ganglion cells were plated on porn/laminin-coated dishes in serum-free culture medium and analyzed after cell attachment. Double-immunostaining showed that all PHOX2B-positive cells co-express the noradrenergic markers TH (**A**) and VMAT1 (**B**) but not the chromaffin cell-specific proteins PNMT (**C**), DLK1 (**D**), and SGII (**E**). ISLET1 (**F**), TUBB3 (**G**), and PERIPHERIN (**H**) expression are shown as examples of neuron-specific proteins. Magnification bar, 60 μm.

Sympathetic neuroblasts were obtained by dissociating E14.5 embryonic mouse sympathetic ganglia (SCG, STG, and thoracic sympathetic chain ganglia). At this stage of development, the vast majority of ganglion cells are proliferating PHOX2B^+^/TH^+^/TUBB3^+^/PERIPHERIN^+^ neuroblasts [59]. Using co-immunostaining with PHOX2B, the sympathetic neuroblasts were shown to express adrenergic- (TH, VMAT1) and pan-neuronal- (TUBB3, ISL1, PERIPHERIN) but not chromaffin cell-specific proteins (PNMT, DLK1, SGII) (Figure 1).

**Figure 2:**
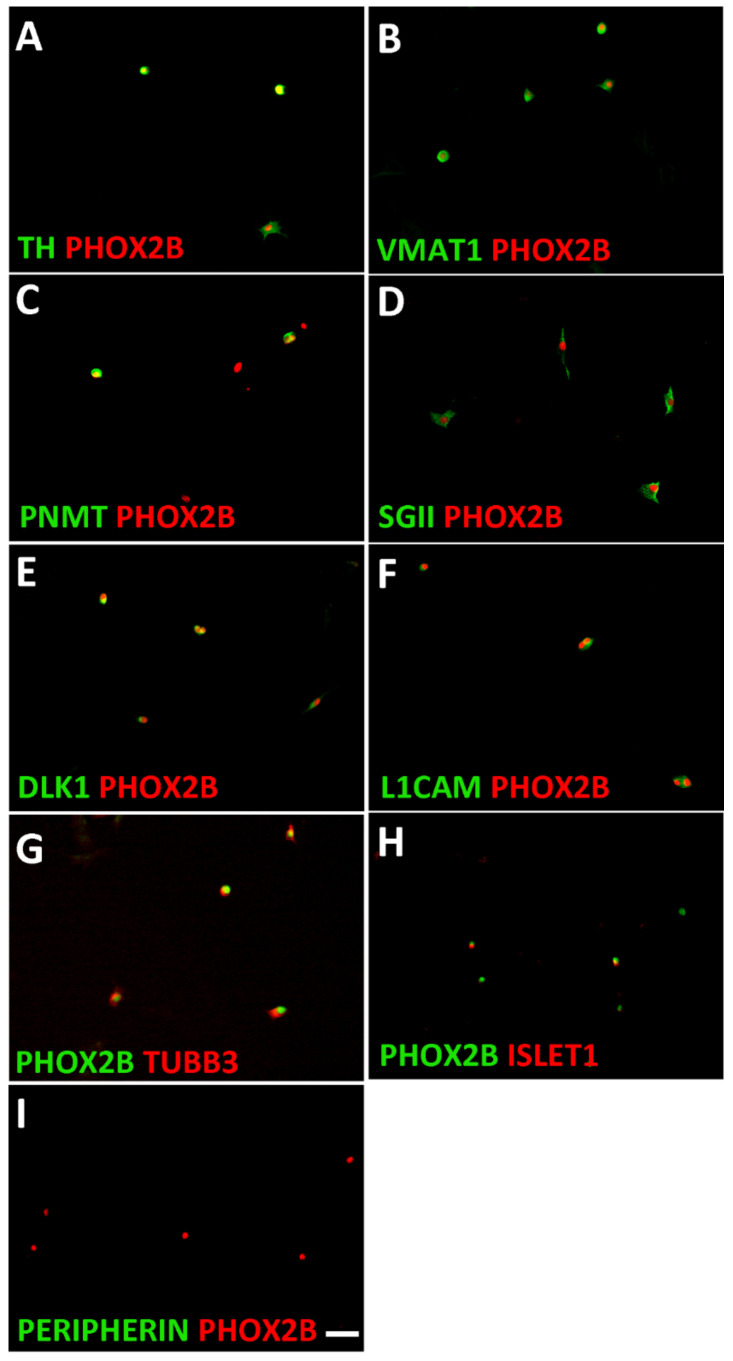
Characterization of mouse adrenal chromaffin cells. Dissociated adrenal cells were plated on porn/laminin-coated dishes in serum-free culture medium and analyzed after cell attachment. Double-immunostaining showed that all PHOX2B-positive cells co-express the noradrenergic proteins TH (**A**) and VMAT1 (**B**). The chromaffin cell-specific proteins PNMT (**C**), SGII (**D**), and DLK1 (**E**) are expressed in 57 ± 3%, 92 ± 2% and 91 ± 4% of PHOX2B-positive cells, respectively. The neuron-specific proteins L1CAM (**F**) and TUBB3 (**G**) are expressed by virtually all chromaffin cells, whereas ISLET1 is only detected in a subpopulation (**H**), and PERIPHERIN is virtually absent in chromaffin cells (**I**). Magnification bar, 60 μm.

As the postnatal adrenal medulla is not only composed of chromaffin cells but also contains a low number of neuroblasts, it is important to define the identity of the cells isolated from sympathetic ganglia and adrenal [8]. Chromaffin cells are characterized by the expression of the pan-autonomic transcription factor PHOX2B, noradrenergic marker proteins such as TH and VMAT1, and genes that are selectively expressed in chromaffin cells but not in sympathetic neurons, such as *Dlk1*, *SgII*, and *Pnmt* [62,63,64]. During development, some pan-neuronal genes are also co-expressed [62]. Using double-immunostaining for PHOX2B as well as for noradrenergic, chromaffin, and neuronal markers, we identified chromaffin cells in dissociated postnatal adrenal as TH^+^/VMAT1^+^/PNMT^+^/SGII^+^/DLK1^+^/L1CAM^+^/TUBB3^+^/ISLET1^+^/PERIPHERIN^−^ cells (Figure 2). PNMT was only detected in 57 ± 3% (mean ± s.e.m.; *n* = 3) of the total chromaffin cells representing the adrenaline-producing A-chromaffin cell subpopulation. The chromaffin cell and neuroblast characterization results are summarized in Table S2.

**Figure 3:**
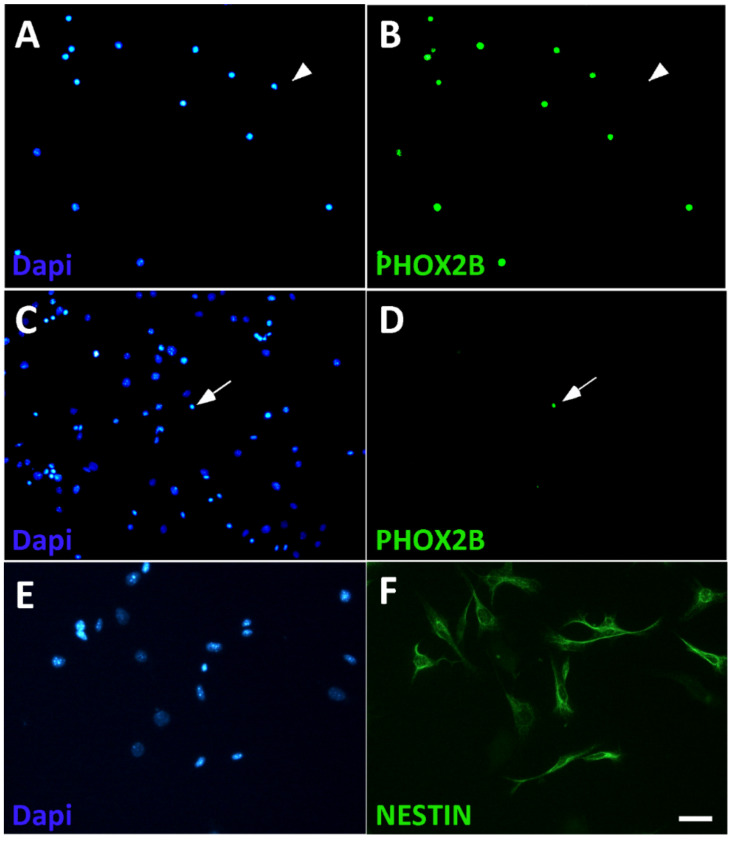
Isolation of chromaffin cells from dissociated adrenal cells by L1CAM panning. L1CAM-positive (**A**,**B**) and L1CAM-negative (**C**,**D**) cell populations were plated after panning and analyzed for the presence of chromaffin cells by co-staining by Dapi for cell nuclei (**A**,**C**) and PHOX2B (**B**,**D**). Most L1CAM-positive cells express PHOX2B (**A**,**B**), whereas the L1CAM-negative population is virtually devoid of PHOX2B-positive cells (**C**,**D**). Arrowheads point to a PHOX2B-negative cell in the L1CAM-positive population (**A**,**B**), arrows point to a PHOX2B-positive cell in the L1CAM-negative population (**C**,**D**). NESTIN expression is detected in most L1CAM-negative cells (**E**,**F**). Magnification bar, 60 μm.

During early postnatal development (P0–P5), chromaffin cell markers were detected in about 10% of the dissociated mouse adrenal cells. The expression of the cell surface protein L1CAM allowed to sort chromaffin cells from adrenal cells via immuno-panning protocols [65]. About 85% of the sorted L1CAM-positive cells were identified as adrenergic cells by the co-expression of PHOX2B and TH, whereas PHOX2B^+^/TH^+^ cells only represent a minority (<2%) of the L1CAM-negative cells (Figure 3). The L1CAM-negative population is heterogenous. NESTIN and S100B are detected in most cells (65 ± 3% and 68 ± 8%, respectively) and are termed as NESTIN-expressing cells (Figure 3 and Figure S1). Minor subpopulations were identified by the expression of SOX10 (6 ± 2%) or STAR (21 ± 2%) (Figure S1). Double-immunostaining revealed that S100B and SOX10 are expressed in NESTIN^+^ cells, whereas the adrenocortical marker STAR was only detected in NESTIN^−^ cells (Figure S1).

**Figure 4:**
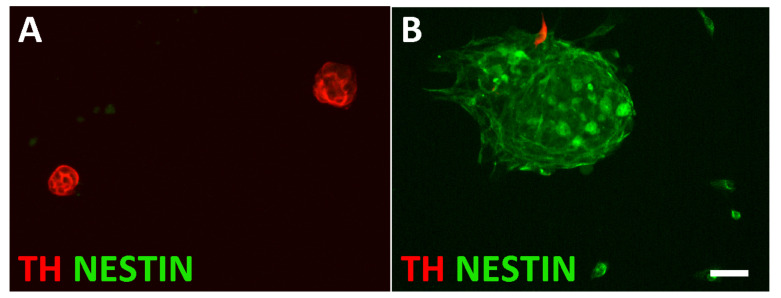
Characterization of sphere cultures from L1CAM-positive and L1CAM-negative cells. L1CAM-positive spheres are composed of TH-expressing cells (**A**), whereas L1CAM-negative spheres are largely composed of NESTIN-positive cells (**B**). Magnification bar, 60 μm.

For further characterization, L1CAM-positive and LCAM-negative cells were expanded in sphere cultures (Figure 4) and analyzed by bulk RNA sequencing (Table S1). The L1CAM-positive spheres showed strong enrichment in the expression of sympathoadrenal genes (*Hand2*, *Phox2b*, *Phox2a*, *Gata2*, *Dbh*) and chromaffin cell marker genes (*SgII, Pnmt*). The expression of pan-neuronal proteins in chromaffin cells (Figure 2) was confirmed by the detection of *Tubb3*, *Nefl*, *Nefm*, *Stmn2*, and *Isl1* mRNA. Notably, peripherin mRNA was not detected in the L1CAM-positive spheres (Table S1).

The L1CAM-negative spheres showed the enhanced expression of genes that were previously described for progenitor cells in mouse, bovine, and human adrenal medulla (*Nestin*, *Snai1*, *Snai2*, *Sox9*, *S100b*) [20,66,67,68]. In addition, genes described for the postnatal mouse adrenal cortex (*Star*, *Cyp11b1*, *Cyp21a1*), and endothelial (*Fabp4*, *Plvap*, *Emcn*, *CD34*) and adrenal capsule cells (*Mgp, Dcn, Col1a1, Ogn*) [8] showed increased expression in the L1CAM-negative population (Table S1).

### 3.2. Signaling Pathways in the Proliferation Control of Neuroblasts and Chromaffin Cells

Having defined the culture conditions for both neuroblasts and chromaffin cells, inhibitors that interfere with the signaling pathways controlling neuroblast proliferation (ALK, WNT, IGF1, PRC2/EZH2) and/or are known to interfere with neuroblastoma growth affecting epigenetic mechanisms (EZH2, BET, CDK7) were investigated. To control for general rather than lineage-specific antiproliferative effects, non-neuronal, non-adrenergic NESTIN-expressing cells were used.

**Figure 5:**
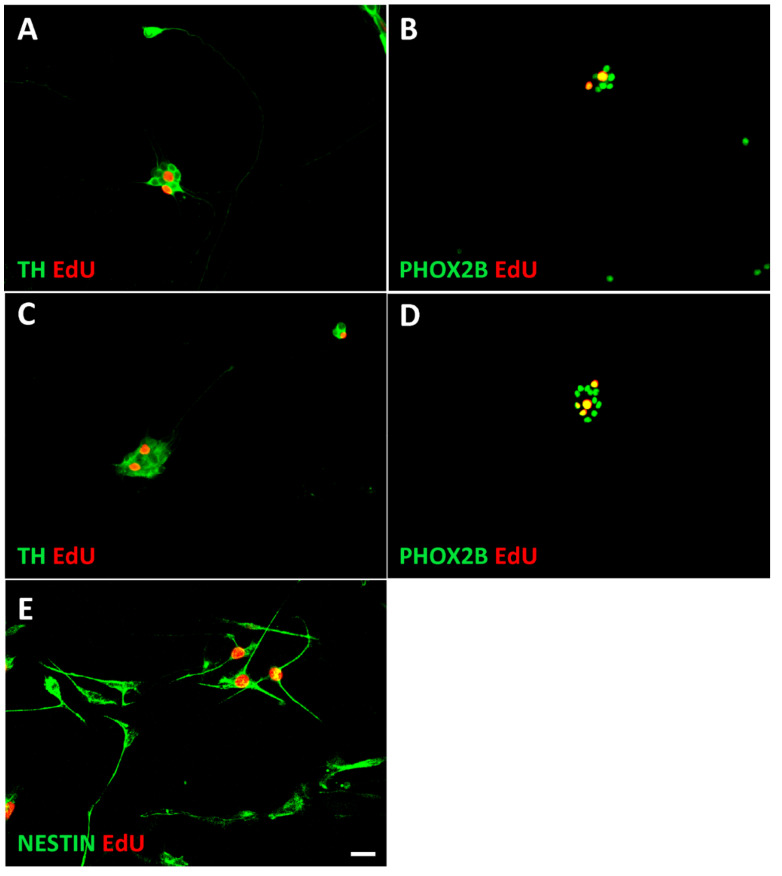
Identification of proliferating neuroblasts, chromaffin cells, and NESTIN-expressing cells. Proliferating cells were identified by a short 4 h EdU pulse, followed by immunostaining for PHOX2B, TH, or NESTIN combined with EdU-staining using the Click-iT protocol. Cultures of sympathetic neuroblasts (**A**,**B**), chromaffin cells (**C**,**D**), and Nestin-expressing cells (NECs) (**E**) are shown. For quantification PHOX2B/EdU rather than TH/EdU staining was used since nuclear staining resulted in better cell identification. Magnification bar, 60 μm.

Substrate-attached rather than sphere cultures were used to allow the unambiguous identification and quantification of individual immunostained cells. Proliferating chromaffin-like cells and sympathetic neuroblasts were identified by a 4-h EdU pulse and co-staining for PHOX2B and EdU (Figure 5). The percentage of proliferating NESTIN-expressing cells was determined by co-staining for NESTIN and EdU (Figure 5).

#### 3.2.1. Similar Effects on Proliferating Neuroblasts, Chromaffin Cells and NESTIN-Expressing Cells by IGFR and EZH2 Inhibition

**Figure 6:**
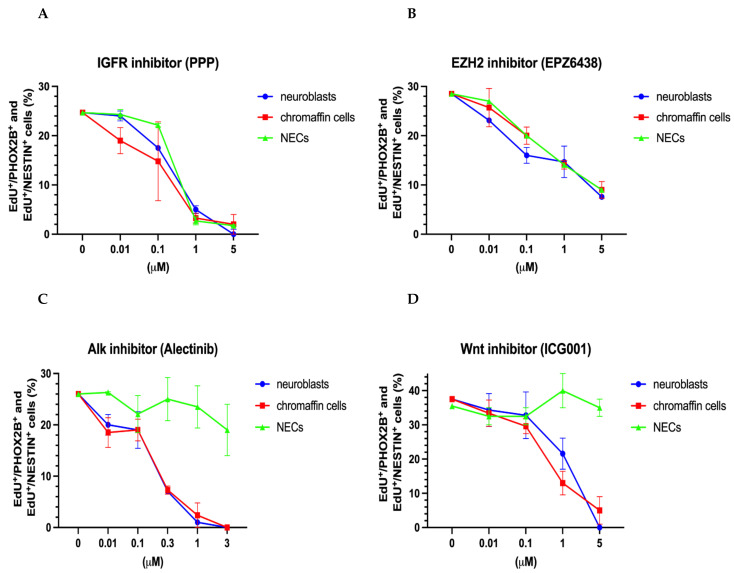
Effect of IGF, EZH2, ALK, and WNT inhibitors on the proliferation of chromaffin cells, neuroblasts, and NESTIN-expressing cells (NECs). Dose–response curves are shown for IGFR inhibitor (PPP) (**A**), EZH2 inhibitor (EPZ6438) (**B**), Alk inhibitor (Alectinib), (**C**) and Wnt inhibitor (ICG001) (**D**). Data represent the mean ± s. e. m. of at least three independent experiments.

To assess the role of the IGF1 signaling pathway and the role of the PRC2 complex, the neuroblasts and chromaffin cells were treated with picropodophyllin (PPP) and EPZ 6438, respectively. PPP blocks IGF1-receptor phosphorylation and downstream signaling and does not interfere with other tyrosine receptor kinases [69]. EPZ-6438 is a potent and selective inhibitor that blocks the histone methyltransferase activity of EZH2 [70]. Our results indicate that neuroblasts, chromaffin cells, and NESTIN-expressing cells have a similar sensitivity to these inhibitors (Figure 6A,B), which suggests a more general, lineage-independent action of the tested molecules.

#### 3.2.2. Common Effects on Proliferating Neuroblasts and Chromaffin Cells by ALK and WNT Inhibition

To document the impact of the ALK and WNT signaling pathways, we used alectinib, a second-generation ALK inhibitor, and the canonical WNT pathway inhibitor ICG001. The effects of the WNT and ALK inhibitors were restricted to sympathoadrenal cells, i.e., neuroblasts and chromaffin cells (Figure 6C,D). As expected, the proliferation of NESTIN-expressing cells was unaffected due to the restriction of ALK expression to the neuroblast lineage that is observed in several species, including humans [6,71]. The impact of the ALK inhibitor alectinib on mouse neuroblast proliferation confirms the ALK-dependence of sympathetic neuroblast proliferation [25,29,30]. Our present results suggest that both neuroblast and chromaffin cell proliferation may involve WNT signaling during normal development. In accordance with the expression of the WNT receptor Fzd5 in chromaffin cells and neuroblasts, but not in SCPs [4], NESTIN-expressing cells were not affected by WNT-inhibition.

#### 3.2.3. Differential Effects of BET and CDK7/12/13 Inhibition on Chromaffin Cells and Neuroblasts

**Figure 7:**
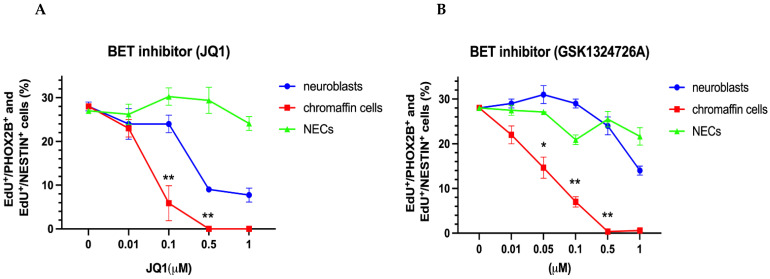

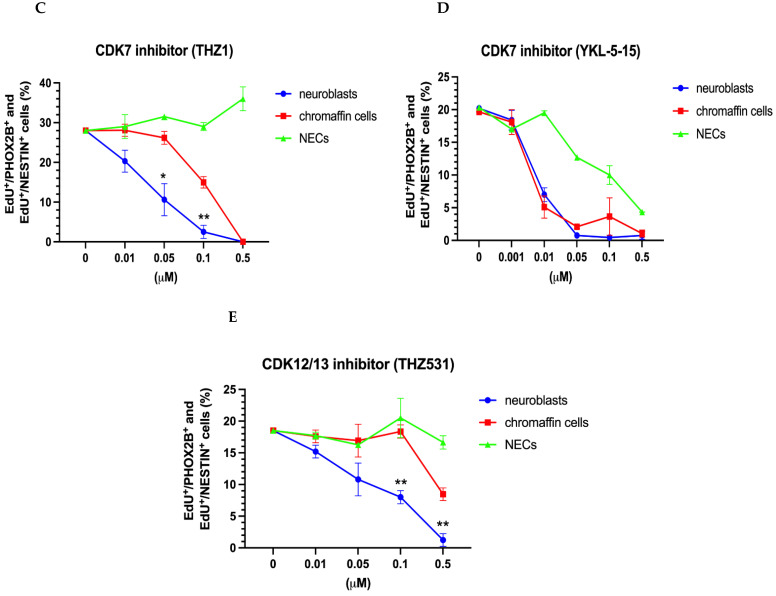
Effect of BET inhibitors JQ1 and GSK1324726A and of CDK inhibitors THZ1, YKL-5-15, and THZ531 on the proliferation of chromaffin cells, neuroblasts, and NESTIN-expressing cells (NECs). Dose–response curves are shown for the BET inhibitors JQ1 (**A**) and CSK1324726A (**B**), CDK7 inhibitors THZ1 (**C**) and YKL-5-15 (**D**) and the CDK12/13 inhibitor THZ531 (**E**), Data represent the mean ± s.e.m. of at least three independent experiments. Significant differences marked by * and ** indicate *p*-values < 0.05 and <0.01, respectively.

We next evaluated the effect of the BET inhibitor JQ1. Interestingly, the neuroblasts, chromaffin cells, and NESTIN-expressing cells differed significantly in their response to JQ1 treatment (Figure 7A). While the proliferation of the NESTIN-expressing cells was unaffected, the chromaffin cells showed a sensitivity to JQ1 inhibition that was ten-fold higher compared to that of the neuroblasts (Figure 7A). The difference in the sensitivity to BET inhibition was even stronger in response to a second BET inhibitor (GSK1324726A/I-BET726) (Figure 7B).

Subsequently, we explored the effects of various CDK inhibitors. THZ1 showed differential effects with stronger neuroblast inhibition compared to chromaffin cell proliferation (Figure 7C). While THZ1 appears to preferentially affect genes controlled by super-enhancers in neuroblastoma, MYCN-dependent genes in particular [44,72], there is evidence to indicate that the off-target inhibition of CDK12 and CDK13 rather than of CDK7 is responsible for the transcriptional effects of THZ1 [72]. To address this issue, we further studied the effects of the selective CDK7 inhibitor YKL-5-124 and the CDK12/13 inhibitor THZ531. Differential effects on neuroblast and chromaffin cell proliferation were observed in response to THZ531 but not YKL-5-125 (Figure 7D,E), which is in agreement with the conclusion that differential THZ1 effects involve CDK12/CDK13 in addition to CDK7 inhibition [72].

Finally, to exclude that the differential effects of BET and CDK inhibitors were related to different culture conditions for neuroblasts and chromaffin cells, inhibitors were applied to chromaffin cells maintained under neuroblast culture conditions (Supplementary Figure S2). The obtained results were fully consistent with those obtained with the standard chromaffin cell cultures.

### 3.3. Plasticity of Chromaffin Cells

During embryonic and postnatal stages, chromaffin cells have the potential to transdifferentiate towards a neuroblast or neuronal phenotype. The neuronal differentiation of cultured postnatal chromaffin cells is known to be induced by FGF, and both FGF and NGF stimulate chromaffin cell proliferation [17,18]. As the serum-free medium used in the present study also contained FGF, we investigated whether the phenotype of the chromaffin cells was altered in culture.

**Figure 8:**
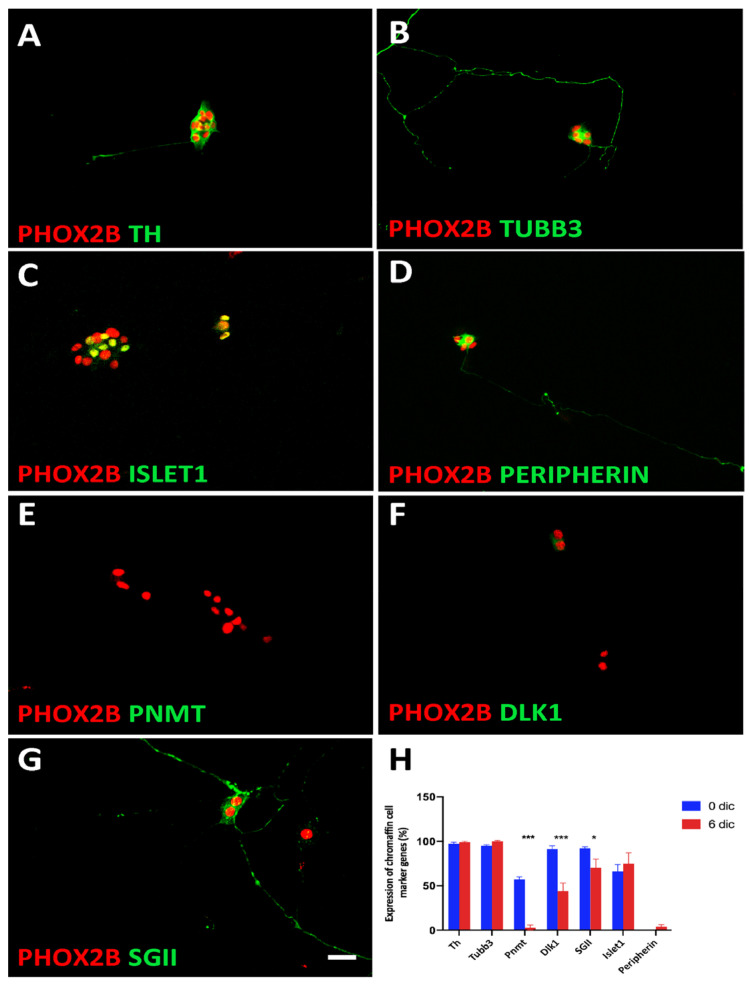
Trans-differentiation of chromaffin cells in culture. Double-immunostaining shows that while TH (**A**) and TUBB3 (**B**) were maintained during the 6d culture period, the chromaffin cell-specific proteins PNMT, DLK1, and SGII are lost or decreased (**E**,**F**,**G**). Increased ISLET1 expression does not reach significance (**C**,**H**). PERIPHERIN is expressed by a very low number of cells both at 3d and 6d in culture (**D**,**H**). The results are quantified in (**H**). Significant differences marked by * and *** indicate *p*-values < 0.05 and <0.001, respectively. Magnification bar, 60 μm.

We observed that a small subpopulation of cells displayed a neuronal phenotype with long neurites after the 6-day-culture period (Figure 8A,B). The neuronal markers that were expressed in the short-term cultures were maintained (TUBB3, ISL1) (Figure 8B,C,H). In parallel, PNMT expression was completely lost (Figure 8E,H), and the expression levels of other chromaffin cell markers were decreased (Figure 8F,G), resulting in a lower percentage of PHOX2B-positive cells expressing DLK1 (44 ± 9%) and SGII (70 ± 12%) compared to chromaffin cells after short-term culture (DLK1 91 ± 4%; SGII 92 ± 4%; PNMT 57 ± 3%) (Figure 8H). The percentage of PHOX2B-positive cells expressing TH and CHGB did not change between 1 and 6 days in culture (97% vs. 100%; 92% vs. 94%). Taken together, our present results reveal partial chromaffin cell dedifferentiation rather than full transdifferentiation to a neuronal identity.

## 4. Discussion

The differences in the clinical outcomes and genetic features between neuroblastoma located in sympathetic ganglia and adrenal are well documented [12,13,14]. We now describe how proliferating mouse chromaffin cells and sympathetic neuroblasts differ in their dependence on BET proteins and CDKs, whereas the inhibition of several other signaling pathways did not result in differential effects. An increased dependence on BET and CDK is expected to result in selective vulnerability to mutations and genomic aberrations affecting BET and CDK-controlled processes. This can explain how different signaling pathways may be involved in tumor initiation and lead to adrenal and ganglionic tumors having distinct characteristics.

### 4.1. Epigenetic Mechanisms: PRC2/EZH2 Inhibitors

The histone methyltransferase EZH2 shows increased expression levels in neuroblastoma and is induced by MYCN binding to the EZH2 promoter [40]. EZH2 inhibition results in the reduced proliferation of neuroblastoma cell lines and decreased tumor growth [73]. The growth inhibition observed for neuroblastoma cells raised the question whether sympathoadrenal cells and NESTIN-expressing cells would respond differentially to EZH2 inhibitors. The similar proliferation effects suggest, however, that global effects on the chromatin structure and gene expression have been targeted that may lead to growth arrest in many cell types.

### 4.2. Epigenetic and Transcriptional Mechanisms: BET and CDK7 Inhibition

Important components of epigenetic regulation are chromatin ”readers” that bind to histone modifications and recruit transcription factors to control the expression of target genes. The members of the BET protein family, BRD2, BRD3, and BRD4, bind to acetylated histone tails and can be targeted by selective inhibitors disrupting this interaction. BRD4 has been shown to be essential for cell identity determination during development via accumulation on a subset of lineage specific super-enhancers, controlling gene transcription as a cofactor of the mediator complex [74,75,76].

BET inhibitors are effective for the treatment of hematopoietic cancer, but neuroblastoma cell lines are also among the cell lines that are the most sensitive to BET inhibition [77]. The cancer-specific susceptibility to BET inhibition has been explained by the acquisition of super-enhancers during tumorigenesis and a preferential loss of BRD4 from the mediator complex at super-enhancers upon treatment with BET inhibitors [43,75]. Initial studies suggested that MYCN amplification is responsible for the increased sensitivity to BET inhibitors, including JQ1 [46]. This notion has been put into question, as the anti-proliferative and cytotoxic effects of the BET inhibitor GSK1324726A (iBET726) did not correlate with MYCN amplification [78]. In addition, although GSK1324726A reduced MYCN expression, the effects could only be partially rescued by MYCN overexpression [78].

Interestingly, cultured mouse chromaffin cells show a sensitivity to BET inhibition caused by JQ1 and GSK132 (EC_50_ ≈ 50 nM), which is in the same range as the one observed for neuroblastoma cell lines, including cell lines with MYCN amplification [46,78]. This may indicate that normal chromaffin cells depend on the activity of a similar set of enhancers as neuroblastoma cells. This notion is supported by the finding that the most active super-enhancers in neuroblastoma cell lines include the enhancers for transcription factors (PHOX2B, HAND2, GATA3) that are essential for the normal development of sympathoadrenal cells and that form a core regulatory circuitry (CRC) in neuroblastoma [79,80,81,82]. In the absence of information on the differences between the super-enhancer landscapes of chromaffin cells and neuroblasts, it remains unclear whether the selective effects of BET inhibition are due to transcriptional effects or whether the pathways controlling genome stability are also affected by BET proteins [77]. Notably, the sensitive response of chromaffin cells to BET inhibition argues against cancer-specific susceptibility and implies that tumor treatment will also affect normally developing cells.

Another approach to interfere with neuroblastoma growth is the inhibition of cyclin-dependent kinase 7 (CDK7) by the covalent inhibitor THZ1 [44]. CDK7 belongs to the class of transcription-associated CDKs, with its main action being the phosphorylation of the Pol II C-terminal domain (CTD) that supports transcriptional initiation, pause release, and elongation [83], although the cell cycle is also affected by the phosphorylation of the key cell cycle CDKs (CDK1, 2 and 4) and other mechanisms [72,84]. CDK7 has recently been shown to act as a master regulator of transcription-associated kinases, directly activating CDKs 9, 12, and 13 [85]. The selective effects of THZ1 on the proliferation and survival of MYCN-amplified neuroblastoma cells were observed in vitro and in vivo [44]. The increased vulnerability of the MYCN-amplified cells compared to tumor cells with normal MYCN levels was explained by high-level transcription from super-enhancer associated-MYCN and other genes with increased Pol II activity. However, the inhibition of CDK7 caused by the more selective CDK7 inhibitor YKL-124 that does not inhibit CDKs 12 and 13 (and displays 100-fold greater selectivity for CDK7 than CDK9 and CDK2) revealed a predominant cell-cycle phenotype with arrest in G1 but no effect on Pol II CTD phosphorylation or Pol II-mediated gene expression [72]. This indicates that the effects of THZ1 on MYCN expression and global transcriptions are due to the combined inhibition of CDK7 and CDK12/13 [72,85].

Compared to chromaffin cells, the cultured sympathetic neuroblasts showed a significantly higher sensitivity to THZ1 with an EC_50_ of about 30 nM, which is between the sensitivity of the neuroblastoma cell lines with and without MYCN amplification [44]. The neuroblasts were also more sensitive to the CDK12/13 inhibitor THZ531 but displayed the same response as the chromaffin cells to the selective CDK7 inhibitor YKL-5124. This suggests that the differential effects of the CDK7 inhibitor THZ1 are mainly due to the off-target inhibition of CDK12/13. Increased vulnerability due to high-level transcription from super-enhancers, which has also been proposed for MYCN-amplified versus non-amplified neuroblastoma cell lines, may be considered as an explanation for the increased neuroblast sensitivity. However, there is at present no obvious candidate for such a difference in the super-enhancer landscape.

Taken together, we showed that BET and CDK7/12/13 differ in their effect on the proliferation of chromaffin cells and neuroblasts, with chromaffin cells being more sensitive to BET inhibitors and neuroblasts being more sensitive to CDK7/12/13 inhibition. Thus, the inhibition of BET and transcriptional CDKs affect different targets in chromaffin cells and neuroblasts. This is in agreement with the finding that JQ1 and THZ1 show synergistic rather than additive effects on the growth and survival of neuroblastoma cell lines [82]. Different mechanisms of action for agents that inhibit transcriptional CDKs and BET-dependent pathways can be expected in view of the many facets of CDK and BET signaling [77,86].

As adrenal neuroblastoma shows a much higher incidence of MYCN amplification and segmental genomic aberrations [12,13,14], it should be noted that the BET pathways include effects on genomic stability. Recent evidence suggests BRD4 functions in DNA damage repair and DNA damage checkpoint that may be linked to cancer development. Increased H4 acetylation at sites of DNA double-strand breaks recruits BRD4 that, in turn, works as a docking site for components of the DNA repair complex [87,88]. BRD4 is also important to prevent transcription–replication conflicts and DNA damage in oncogene-driven cells [48]. Interestingly, the induction of DNA damage by BET inhibition varies across cancer cell lines, which may be due to different basal levels of replication stress [48,89]. The increased vulnerability of chromaffin cells to BET inhibition may thus indicate increased replication stress resulting in a higher probability of genomic instability leading to chromosomal alterations characteristics for adrenal neuroblastoma.

The hypothesis of a sympathoadrenal progenitor as the origin of both chromaffin cells and sympathetic neurons was derived from the observation that postnatal chromaffin cells can be directed towards a neuron or endocrine fate by extrinsic signals [2,3]. In the present study, the plasticity of chromaffin cells is also shown by the loss or decreased expression of chromaffin cell specific genes. The absence of PNMT is characteristic for noradrenergic N-chromaffin cells. The decrease in DLK1 and SGII expression suggests that chromaffin cells may eventually transdifferentiate towards an adrenal neuroblast identity. This plasticity has implications for the concepts of adrenal neuroblastoma initiation, raising the possibility that malignant adrenal neuroblastoma cells may originate from transdifferentiating chromaffin cells rather than from embryonic adrenal neuroblasts. The increased dependence of normal chromaffin cells on the BET pathways may affect their response to oncogenic aberrations and result in adrenal-specific tumor characteristics.

## 5. Conclusions

Chromaffin cells and neuroblasts differ in their sensitivity to BET and CDK inhibition. This finding supports the notion of different founder cells, chromaffin cells, and neuroblasts that differ in their vulnerability to oncogenic signals at the origin of adrenal and ganglionic neuroblastoma.

## Data Availability

Raw data for RNA-seq are available in the Gene Expression Omnibus (GEO) under the accession number GSE200379 (https://www.ncbi.nlm.nih.gov/geo/query/acc.cgi?acc=GSE200379).

## Supplementary Materials

The following are available online at https://www.mdpi.com/article/10.3390/cancers14112755/s1, Figure S1: Characterization of L1CAM-negative cell population; Figure S2: Differential sensitivity of chromaffin cells and neuroblasts to BET and CDK inhibitors is independent of culture conditions; Table S1: Differential gene expression of adrenal L1CAM-positive and L1CAM-negative cells analyzed by bulk RNA sequencing; Table S2: Characterization of sympathetic neuroblasts and chromaffin cells.
