# Supplementary material for: BET and CDK Inhibition Reveal Differences in the Proliferation Control of Sympathetic Ganglion Neuroblasts and Adrenal Chromaffin Cells
Source: Cancers (Basel). 2022 Jun 1;14(11):2755. doi: 10.3390/cancers14112755 (PMC9179499; doi:10.3390/cancers14112755)
Supplement: Supplementary file 1 [file cancers-14-02755-s001.zip › cancers-1731474-supplementary/cancers-1731474 Supplementary Materials-done_HR.pdf]

cancers MDPI

Supplementary Materials: BET and CDK Inhibition Reveal Differences in the Proliferation Control of Sympathetic Ganglion Neuroblasts and Adrenal Chromaffin Cells

Jessica Sriha, Caroline Louis-Brennetot, Cécile Pierre-Eugène, Sylvain Baulande, Virginie Raynal, Amira Kramdi, Igor Adameyko, Uwe Ernsberger, Thomas Deller, Olivier Delattre, Isabelle Janoueix-Lerosey, and Hermann Rohrer

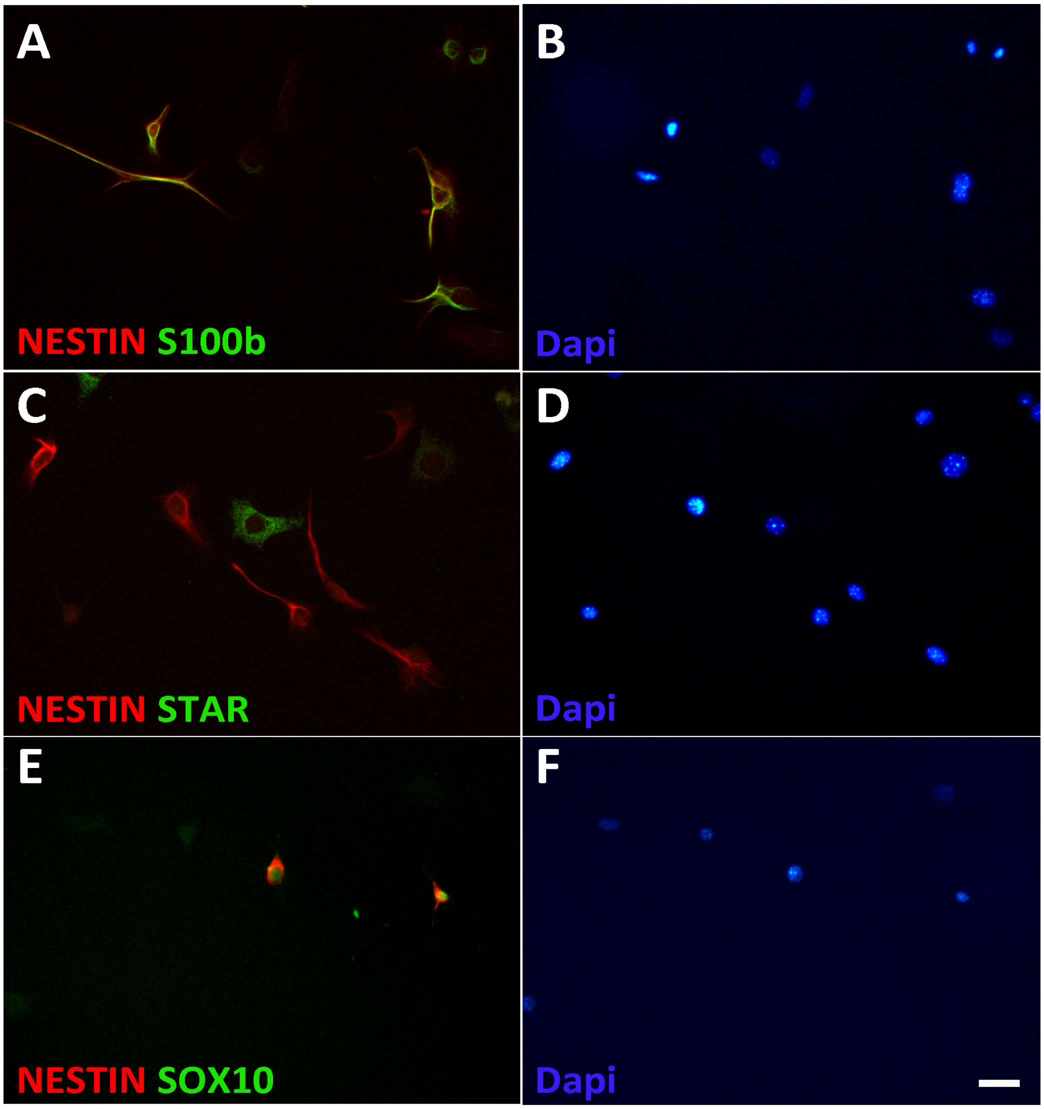

Figure S1. Characterization of the L1CAM-negative cell population. L1CAM-negative cells were plated on Poly-DL-ornithine/laminin substrate in complete serum-free medium. After attachment, cells were fixed and double-stained with anti-NESTIN and anti-S100b (**A**), anti-NESTIN and anti-STAR (**C**), anti-NESTIN and anti-SOX10 (**E**). Nuclei are stained by Dapi (**B,D,F**). Magnification bar 60µm.

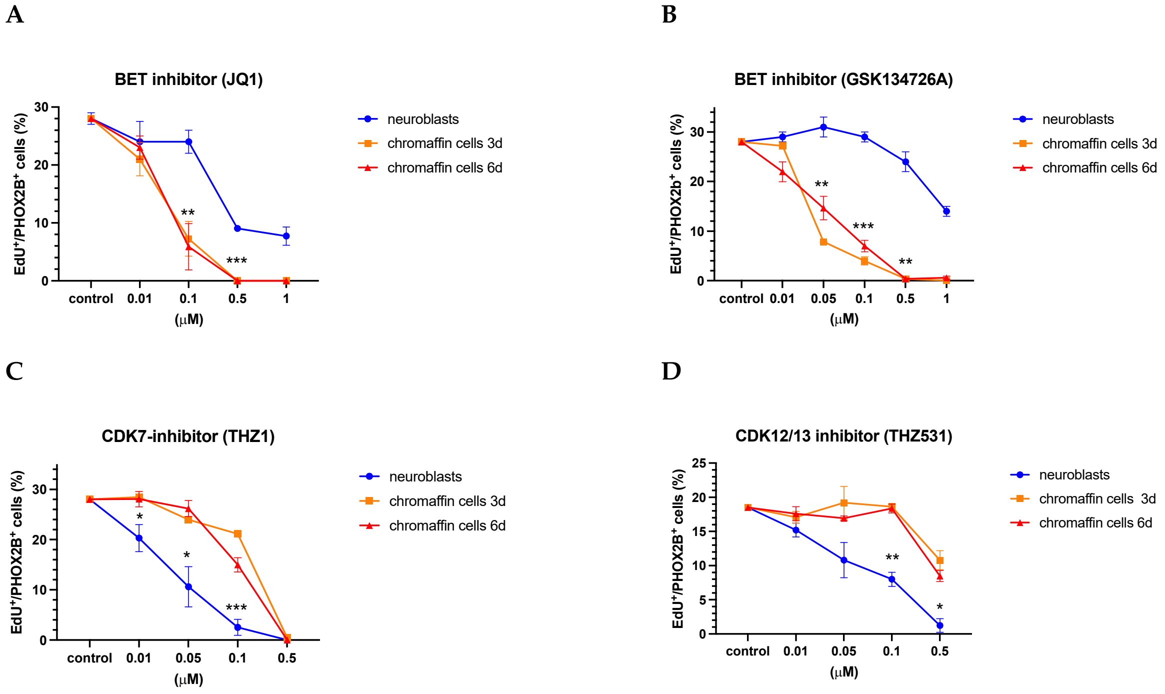

Figure S2. Differential sensitivity of chromaffin cells and neuroblasts to BET and CDK inhibitors is independent of culture conditions. Chromaffin cells maintained either in complete serum-free medium for 6 days (red) or in medium modified for neuroblast culture for 3 days (orange) show similar dose-response to treatment with JQ1 (**A**), GSK134726A (**B**), THZ1 (**C**) and THZ531 (**D**) and differ from neuroblast cultures (blue) (3d). Significant differences between 3 days neuroblast and 3 days chromaffin cell cultures are indicated (* P <0.05; ** P < 0.01; *** P <0.001).

Table S1. Differential gene expression of adrenal L1CAM-positive and L1CAM-negative cells analyzed by bulk RNA sequencing (separate Excel file). List of the Top 500 genes more expressed in L1CAM-positive cells (blue) or in L1CAM-negative cells (green). For each gene ID, median expression in L1CAM-negative cells, in L1CAM-positive cells, fold change (log2), chromosome start and end position, and gene symbol are provided.

Table S2. Characterization of sympathetic neuroblasts and chromaffin cells.

Marker Proteins		Neuroblasts	Chromaffin Cells
autonomic	PHOX2B	+	+
noradrenergic	TH	+	+
	VMAT1	+	+
neuronal	TUBB3	+	+
	ISLET1	+	+ *
	L1CAM	+	+
	PERIPHERIN	+	-
chromaffin cell-specific	PNMT	-	+ **
	DLK1	-	+
	SGII	-	+
progenitor	NESTIN	-	-
	S100B	-	-

The identification of developing sympathetic neuroblasts and chromaffin cells by the analysis of characteristic marker proteins is summarized. Neuroblasts and chromaffin cells express autonomic (PHOX2B), noradrenergic (TH, VMAT1) and several neuronal proteins (TUBB3, ISLET1, L1CAM) but are distinguished by the lack of PERIPHERIN expression in chromaffin cells and the absence of chromaffin cell-specific proteins PNMT, DLK1 and SGII in neuroblasts. Neuroblasts and chromaffin cells do not express the progenitor proteins NESTIN and S100B; * ISLET1 and ** PNMT are expressed by 66 ± 8% and 57 ± 3% of PHOX2B-positive cells, respectively.
